# Germline and conditional ghrelin knockout increases islet size

**DOI:** 10.1172/JCI175799

**Published:** 2023-12-15

**Authors:** Sean M. Tatum, William L. Holland

**Affiliations:** Department of Nutrition and Integrative Physiology and the Diabetes and Metabolism Research Center, University of Utah, Salt Lake City, Utah, USA.

## Abstract

Conflicting studies in recent years report that genetic or pharmacological increases or decreases in ghrelin either increase or have no effect on islet size. In this issue of the *JCI*, Gupta, Burstein, and colleagues applied a rigorous approach to determine the effects of reducing ghrelin on islet size in germline and conditional ghrelin-knockout mice as well as across varying ages and weight. Both germline and conditional ghrelin-knockout mice associated with increased islet size, which was further exacerbated by older age and diet-induced obesity. These findings suggest that modulation of ghrelin may open a therapeutic window to prevent or treat diabetes.

## Ghrelin and diabetes

Ghrelin’s ability to increase body weight by stimulating appetite and augmenting food intake and to increase secretion of growth hormone is mainly mediated by its binding to the CNS and pituitary growth hormone secretagogue receptors (GHSRs) (1). The presence of these receptors in all four pancreatic endocrine cell populations (α, β, γ, and δ) suggests that ghrelin also has potent hormonal effects to maintain blood glucose (2). Indeed, ghrelin counteracts hypoglycemia by suppressing insulin secretion while increasing glucagon secretion (2). In addition to its glucoregulatory effects, Gupta, Burstein, and co-authors provide evidence in this issue of the *JCI* that ghrelin dictates pancreatic and, more specifically, islet size, in turn influencing islet function (3).

**Figure 1:**
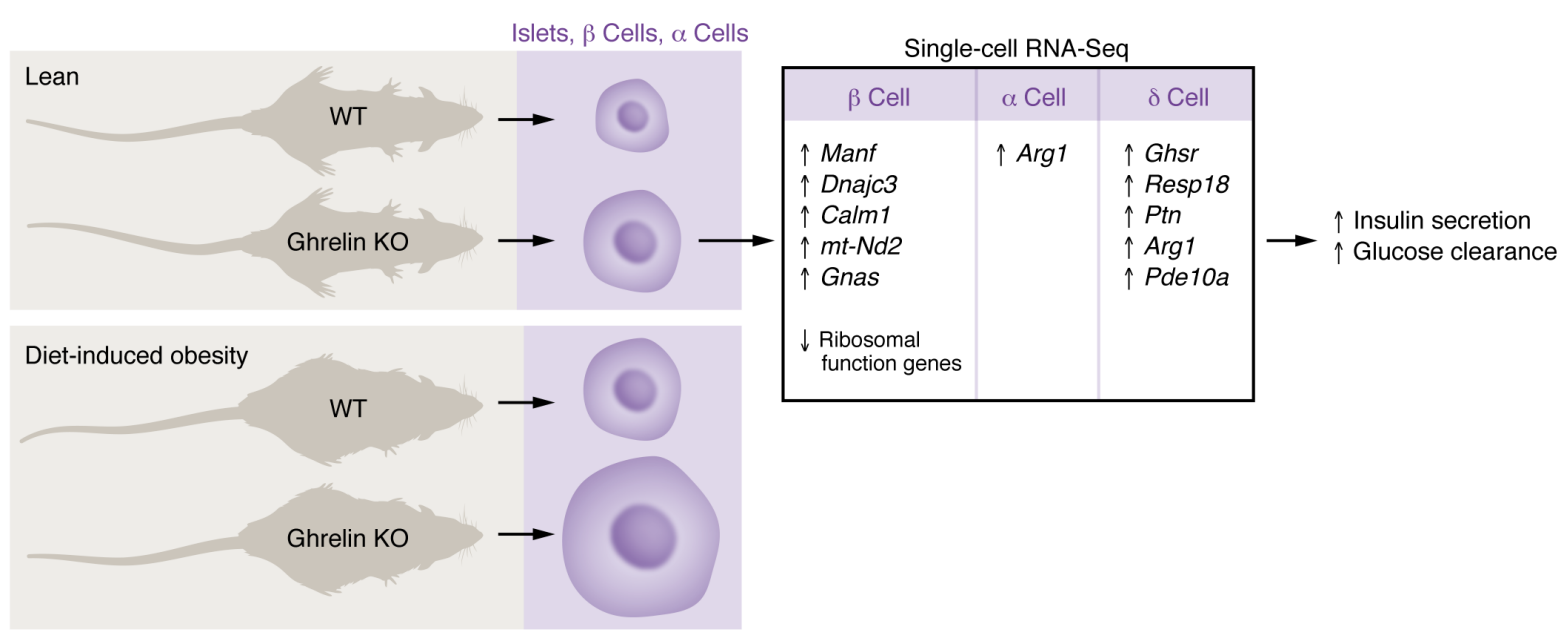
Ghrelin affects pancreatic cell size. Lean (juvenile and adult) and obese ghrelin cell–knockout mice develop hypertrophied islets, β cells, and α cells. Single-cell RNA-Seq suggests that a subset of differentially expressed genes within the islet lead to elevated insulin secretion and glucose clearance.

Patients with obesity often present with decreased plasma levels of ghrelin (4) and greater pancreatic volume (5), corroborating the findings from Gupta, Burstein, and co-authors. Plasma ghrelin levels in patients with diabetes mellitus (DM) are not nearly as clear-cut and may depend on the etiology and stage of diabetes. For example, plasma ghrelin levels are higher in patients with maturity-onset diabetes of the young (MODY) as compared with patients with type 1 or type 2 DM (6). This islet-colocalized expression of ghrelin, coupled with the presence of GHSRs, suggests that ghrelin mediates pancreatic islet form and function. Indeed, studies in perfused rat pancreas demonstrate that ghrelin is secreted from islets (7). Moreover, neutralizing the acute actions of ghrelin enhances glucose-stimulated insulin secretion from perfused pancreas or isolated islets (8). Further evidence that ghrelin governs pancreatic development is found in fetal islets, where ghrelin-secreting ε cells are present (9). Using the power of mouse genetics to allow for the loss of ghrelin in the developing mouse or adult mouse, the Zigman laboratory has established ghrelin as a regulator of islet mass (3) (Figure 1).

## Ghrelin-knockout mice display increased islet size

In this issue of the *JCI*, Gupta, Burstein, and co-authors demonstrated that reducing ghrelin, by germline or conditional deletion of ghrelin cells in juvenile and adult mice increased islet size, the percentage of very large islets, and β cell cross-sectional area and improved glucose tolerance through increased insulin secretion. The increased islet size as a result of ghrelin knockout was observed in mice at four weeks of age and became more pronounced at 10 to 12 weeks of age but was not apparent in neonatal mice, suggesting that increased islet size could occur when ghrelin is absent during early development. In fact, ghrelin-knockout mice challenged with a high-fat diet (HFD) had larger islet sizes and greater numbers of very large islets compared with WT mice challenged with a HFD, suggesting that decreased ghrelin was not solely responsible for islet and β cell enlargement. The authors stated that elevated β cell numbers in ghrelin-knockout mice was likely mediated by reduced β cell apoptosis as opposed to proliferation. Ghrelin-knockout mice also exhibited an increase in α cell cross-sectional area, further highlighting ghrelin’s ability to regulate islet cell abundance.

To unveil potential molecular mediators through which decreased ghrelin results in islet proliferation, Gupta, Burstein, and co-authors performed single-cell RNA-Seq of adult ghrelin-knockout mice. The four traditional islet endocrine cell types were those clusters with the lowest numbers of differentially expressed genes due to ghrelin deletion. Nevertheless, within β cells, *Manf*, *Dnajc3*, *Calm1*, *mt-Nd2*, and *Gnas* were among the most highly upregulated genes, while many ribosomal function genes were downregulated. Interestingly, ghrelin deletion did not alter the expression of *Ghsr* or the genes encoding the four main islet hormones. However, within δ cells, in which *Ghsr* expression was highest within islet cell clusters, *Resp18*, *Ptn*, and *Arg1* were among the most highly upregulated in response to ghrelin deletion. *Arg1* was also upregulated in α cells. Interestingly, activated stellate cells and endothelial cells exhibited the highest number of differentially expressed genes per cluster size in response to ghrelin deletion. While these single-cell RNA-Seq data do not provide a specific mechanism, they suggest that ghrelin has distinct transcriptional influence on each cell type within the islet. Moreover, these data suggest that ghrelin deletion influences not only gene expression within the four hormone-secreting cells within the islet, but also alters gene expression in other cell types within the islet. Thus, paracrine factors within the pancreas may contribute to the overall effect of ghrelin on glucoregulation. These data open the door for further investigation into the mechanism of how ghrelin regulates islet size and function.

## Implications and conclusions

To our knowledge, this is the first study to report that ghrelin deletion alone was sufficient to increase islet size and β cell mass. These findings are potentially controversial, as prior studies had only described increased islet area upon reduction of ghrelin in conjunction with leptin deficiency (10). Additionally, other groups have found no effect on islet cells in ghrelin deletion (7), GHSR deletion (11), GHSR inhibition (12), or exogenous ghrelin administration (13). On the contrary, Gupta, Burstein, and colleagues corroborated previous findings by demonstrating no effect of ghrelin on islet size at embryonic stages. The authors surmise that the rigor with which they performed their studies by using different ages and dietary challenges sheds a light on the role of ghrelin in islet size compared with previous studies.

The observation that conditional knockout of ghrelin induces intrigue into the possible therapeutic ability of ghrelin reduction to increase β cell mass in patients with type 1 DM. The authors postulate that neutralization of ghrelin or inhibition of GHSR signaling could be used to increase β cell numbers for these patients as well as those undergoing pancreatic transplantation. Inhibition of ghrelin action may also aid type 2 DM management by preventing β cell apoptosis. However, in the context of type 2 DM, a concern arises insofar as ghrelin-knockout mice challenged with a HFD presented with larger islets than did WT mice challenged with a HFD. It remains possible that the role of ghrelin in the islet may not involve mediating islet size in the context of type 2 DM. Nevertheless, the data presented here warrant further studies of the potential therapeutic utility of ghrelin inhibition to treat diabetes. Additionally, further studies are necessary to determine the exact role for ghrelin with regard to specific diabetes subtypes, islet size, and function and which cell types mediate ghrelin’s effects.

Last, one large philosophical question remains: What is the evolutionary benefit of ghrelin depletion to islet and β cell size?

## Version 1 12/15/2023

Electronic publication
